# Association of high-sensitivity C-reactive protein and odds of breast cancer by molecular subtype: analysis of the MEND study

**DOI:** 10.18632/oncotarget.27991

**Published:** 2021-06-22

**Authors:** Anjali Gupta, Taofik Oyekunle, Omolola Salako, Adetola Daramola, Olusegun Alatise, Gabriel Ogun, Adewale Adeniyi, April Deveaux, Veeral Saraiya, Allison Hall, Omobolaji Ayandipo, Thomas Olajide, Olalekan Olasehinde, Olukayode Arowolo, Adewale Adisa, Oludolapo Afuwape, Aralola Olusanya, Aderemi Adegoke, Trygve O. Tollefsbol, Donna Arnett, Michael J. Muehlbauer, Christopher B. Newgard, H3 Africa Kidney Research Network, Tomi Akinyemiju

**Affiliations:** ^1^Trinity College of Arts and Sciences, Duke University, Durham, NC, USA; ^2^Department of Population Health Sciences, School of Medicine, Duke University, Durham, NC, USA; ^3^College of Medicine & Lagos University Teaching Hospital, University of Lagos, Lagos State, Nigeria; ^4^Obafemi Awolowo University Teaching Hospital, Ile-Ife, Osun State, Nigeria; ^5^University College Hospital, University of Ibadan, Ibadan, Oyo State, Nigeria; ^6^Federal Medical Center, Abeokuta, Ogun State, Nigeria; ^7^Department of Epidemiology, UNC Gillings School of Global Public Health, Chapel Hill, NC, USA; ^8^Department of Pathology, School of Medicine, Duke University, Durham, NC, USA; ^9^Our Lady of Apostle Catholic Hospital, Ibadan, Oyo State, Nigeria; ^10^Department of Biology, College of Arts and Sciences, University of Alabama at Birmingham, AL, USA; ^11^College of Public Health, University of Kentucky, Lexington, KY, USA; ^12^Duke Molecular Physiology Institute, Duke University, Durham, NC, USA; ^13^Department of Internal Medicine, School of Medicine, University of Kansas Medical Center, Kansas City, KS, USA; ^14^Duke Cancer Institute, School of Medicine, Duke University, Durham, NC, USA; ^15^Duke Global Health Institute, Duke University, Durham, NC, USA

**Keywords:** C-reactive protein, breast cancer, Nigeria, molecular subtype, menopausal status

## Abstract

Breast cancer (BC) in Nigeria is characterized by disproportionately aggressive molecular subtypes. C-reactive protein (CRP) is associated with risk and aggressiveness for several types of cancer. We examined the association of high-sensitivity CRP (hsCRP) with odds of BC by molecular subtype among Nigerian women. Among 296 newly diagnosed BC cases and 259 healthy controls, multivariable logistic regression models were used to estimate adjusted odds ratios (aOR) and 95% confidence intervals (CI) for the association between hsCRP and odds of BC overall and by molecular subtype (luminal A, luminal B, HER2-enriched and triple-negative or TNBC). High hsCRP (> 3 mg/L) was observed in 57% of cases and 31% of controls and was associated with 4 times the odds of BC (aOR: 4.43; 95% CI: 2.56, 7.66) after adjusting for socio-demographic, reproductive, and clinical variables. This association persisted regardless of menopausal status and body mass index (BMI) category. High hsCRP was associated with increased odds of TNBC (aOR: 3.32; 95% CI: 1.07, 10.35), luminal A BC (aOR: 4.03; 95% CI: 1.29, 12.64), and HER2-enriched BC (aOR: 6.27; 95% CI: 1.69, 23.25). Future studies are necessary in this population to further evaluate a potential role for CRP as a predictive biomarker for BC.

## INTRODUCTION

In 2018, there were over 2 million cases and 0.6 million deaths from breast cancer (BC), making it the most common cancer globally among women [1]. The past few decades have seen rising BC incidence rates on the African continent [2], with the highest age-standardized mortality rate within the continent in Nigeria [3]. BC in Nigeria is characterized by disproportionately aggressive molecular subtypes, with exceptionally high rates of triple-negative (TN) BC [4], similar to BC in other countries in West Africa [5] and among African American women in the United States [6]. TNBCs are estrogen (ER), progesterone (PR), and human epidermal growth factor-2 (HER2) receptor negative, and less responsive to treatment compared to less aggressive subtypes, leading to poor clinical outcomes [7]. The complex associations of genetic, environmental and lifestyle factors contributing to aggressive BC subtypes are areas of active research [8], and recent studies suggest that chronic inflammation, involving both innate and adaptive immunity, may contribute to tumor heterogeneity and aggressiveness [9]. Despite the well-documented patterns of late stage, distant metastasis and TN subtype tumors among African women, no study to our knowledge has directly examined biomarkers of chronic inflammation in relation to odds of BC and molecular subtypes in Nigerian women.

C-reactive protein (CRP) is an easily measurable biomarker that reflects systemic inflammation, infection, or tissue damage in the body, and may be elevated in both acute and chronic conditions [10, 11]. Circulating levels of CRP have been shown to be elevated in various types of cancers from case-control or cross-sectional studies [12]. In addition, circulating levels of CRP have also been associated with tumor prognosis in patients with several types of solid cancers [13]. Many past studies in the United States, Europe, and Asia have evaluated the association between CRP levels in the blood and BC risk. One systematic review reported a significant positive association between elevated levels of CRP and risk of BC, noting that geographic region might be a possible source of heterogeneity in results, with stronger associations observed among participants from Asia [14]. Another systematic review found no strong evidence for an association between circulating CRP and BC risk among prospective studies [12], while a third systematic review observed a modest but significant positive association [15]. Analyses of the Women’s Health Study found that baseline CRP level was not associated with risk of invasive BC during 10 years of follow-up [16, 17], however, in the Women’s Health Initiative, pre-diagnostic CRP was associated with an increased BC risk among lean women, whereas no association was observed among overweight-obese women [18]. On the contrary, another study in Europe found a positive association between CRP levels and postmenopausal BC risk restricted to women with excess adiposity [19]. Further research on this topic is needed to clarify this relationship. Additionally, it is worth noting that most of these past studies have been conducted in populations from the United States and Europe, among mostly White study populations, and to our knowledge, none have been conducted in populations from Africa.

Notably, few epidemiological studies have analyzed the relationship between CRP levels and BC by molecular subtype, and results have been conflicting. One study in Italy reported a significant association between high CRP and TNBC and luminal B premenopausal BC [20], while another study in China found an association only for hormone receptor positive and HER2 negative BC [21]. Ours is the first study, to our knowledge, to evaluate this relationship on the African continent, where TNBC prevalence is notably higher. Because CRP is an easily measurable biomarker, further insight on this association may elucidate its potential as a simple and cost-effective method for predicting future risk of BC, and as an additional prognostic predictor for survival among BC patients.

## RESULTS

**Figure 1:**
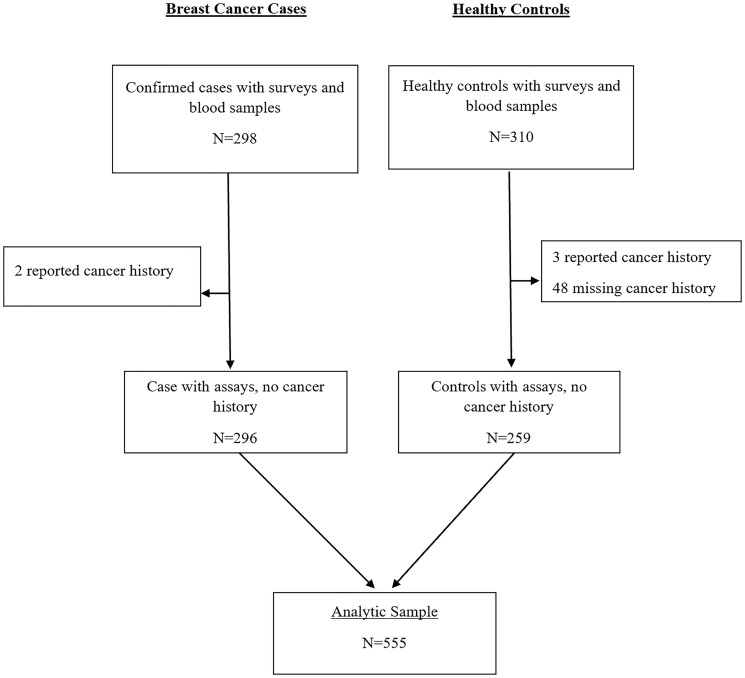
CONSORT diagram for MEND hsCRP analysis.

**Figure 2:**
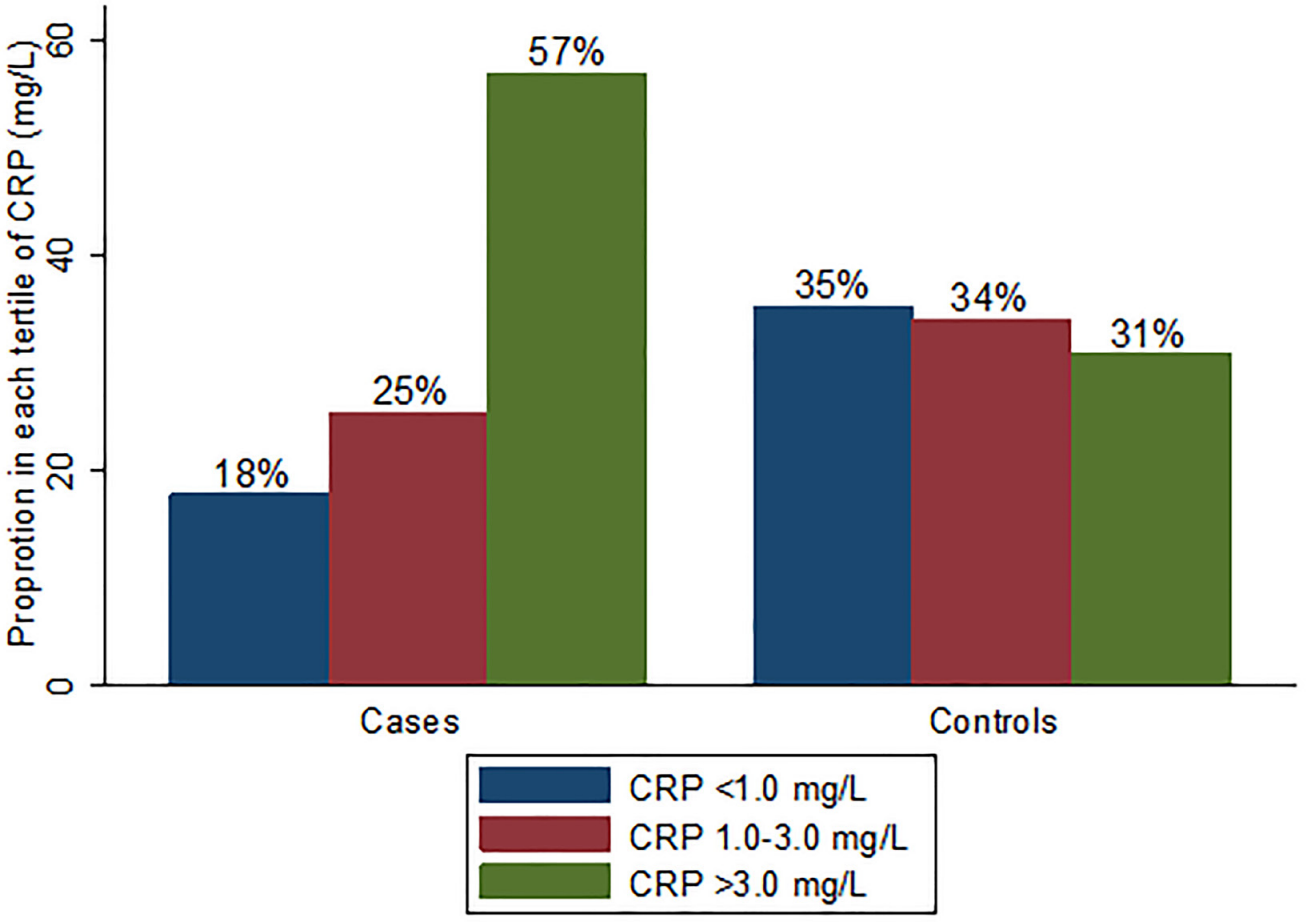
Proportion in each hsCRP category by case-control group.

**Figure 3:**
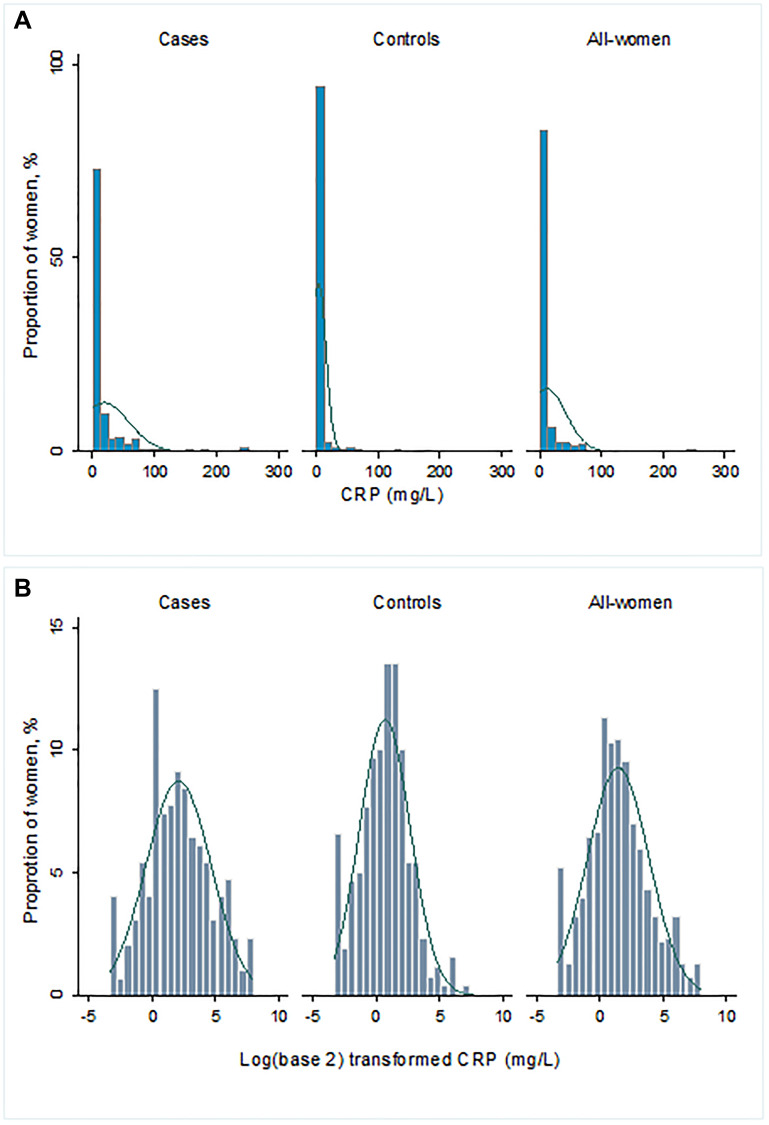
(**A**) Distribution of hsCRP (mg/L) and (**B**) log_2_-transformed hsCRP (mg/L).

Of the 555 women included in the study cohort, 296 (53%) were confirmed BC cases, and 259 (47%) were controls (Figure 1). Compared to controls, cases had higher high-sensitivity CRP (hsCRP) (median 3.9 vs. 1.8 mg/L, *p* < 0.001) (Table 1; Figures 2 and 3). Cases were also more likely to report prior diagnosis of diabetes (26.0% vs. 15.1%, *p* < 0.001), hypertension (18.9% vs. 48.3%, *p* < 0.001) and past use of hormone replacement therapy compared with controls (0.7% vs. 15.1%; *p* < 0.001). No statistically significant differences were found between cases and controls on age at enrollment, age at menarche, number of pregnancies and number of live births (all *p*-value ≥ 0.05). Across hsCRP categories, no statistically significant differences were found between cases and controls in age at enrollment, body mass index (BMI), menopausal status, number of pregnancies and number of live births (all *p*-value ≥ 0.05) (Supplementary Table 1).

**Table 1:** Clinical and reproductive characteristics among breast cancer cases and controls

	Case (*N* = 296)	Control (*N* = 259)	Total (*N* = 555)	*p* value
Demographics				
Age (years)^a^	48 (23–85)	49 (18–74)	49 (18–85)	0.632^1^
Clinical characteristics				
hsCRP (mg/L)^a^	3.9 (0.1–252.0)	1.8 (0.1–129.9)	2.4 (0.1–252.0)	< 0.001^1^
hsCRP (mg/L), categorical				< 0.001^2^
< 1.0	53 (17.9%)	91 (35.1%)	144 (25.9%)	
1.0–3.0	75 (25.3%)	88 (34.0%)	163 (29.4%)	
> 3.0	168 (56.8%)	80 (30.9%)	248 (44.7%)	
BMI (kg/m^2^)				0.086^2^
Underweight	16 (5.4%)	6 (2.3%)	22 (3.9%)	
Normal weight	120 (40.5%)	93 (35.9%)	213 (38.4%)	
Overweight	88 (29.7%)	79 (30.5%)	167 (30.1%)	
Obese	65 (22.0%)	81 (31.3%)	146 (26.3%)	
Height, cm^a^	63.1 (56.1–70.1)	63.0 (51.8–69.5)	63.0 (51.8–70.1)	0.205^1^
Weight, kg^a^	143.0 (81.6–255.2)	149.5 (78.9–289.7)	145.2 (78.9–289.7)	0.019^1^
Systolic BP^a^	125.0 (84.0–236.0)	127.7 (77.7–231.3)	126.7 (77.7–236.0)	0.407^1^
Diastolic BP^a^	79.7 (41.0–136.0)	76.7 (35.3–128.7)	78.0 (35.3–136.0)	0.215^1^
Prior diabetes diagnosis	77 (26.0%)	39 (15.1%)	116 (20.9%)	< 0.001^2^
Prior hypertension diagnosis	56 (18.9%)	125 (48.3%)	181 (32.6%)	< 0.001^2^
Reproductive history				
Age at menarche^a^	15 (9–22)	15 (10–28)	15 (9–28)	0.507^1^
Ever pregnant	282 (95.3%)	243 (93.8%)	525 (94.6%)	0.500^2^
Number of pregnancies^a,b^	5 (1–11)	5 (1–14)	5 (1–14)	0.965^1^
Number of births^a,b^	4 (0–10)	4 (0–16)	4 (0–16)	0.523^1^
Menopausal status				0.504^2^
Pre- or peri-menopause	143 (48.3%)	109 (42.1%)	252 (45.4%)	
Post-menopause	153 (51.7%)	131 (50.6%)	284 (51.2%)	
Ever used HRT	2 (0.7%)	39 (15.1%)	41 (7.4%)	< 0.001^2^

^1^Wilcoxon rank sum test, ^2^Chi-Square test. ^a^Median (range); ^b^Among those who were ever pregnant. Where applicable, missing values were not used in generating *p*-value.

High hsCRP (> 3.0 mg/L) was associated with increased odds of BC in unadjusted models (OR: 3.61; 95% CI: 2.34, 5.55), age-adjusted models (aOR: 3.60; 95% CI: 2.34, 5.54) and in models adjusting for age and reproductive variables (aOR: 4.46; 95% CI: 2.70, 7.38) (Table 2). After additionally adjusting for BMI, diabetes, and hypertension, there was an almost 5-fold increased odds of BC (aOR: 4.43, 95% CI: 2.56, 7.66). Doubling of hsCRP concentration was associated with about 30% increased odds of BC (aOR: 1.31, 95% CI: 1.19, 1.45) in models adjusting for age, BMI, diabetes, hypertension, and reproductive variables. Regardless of menopausal status, high hsCRP remained associated with increased odds of BC in all models considered. In BMI stratified analyses, high hsCRP was associated with increased odds of BC regardless of BMI status (Table 3).

**Table 2:** Multivariable associations of hsCRP and breast cancer by menopausal status

	n/N	Model 1^a^ OR (95% CI)	Model 2^b^ aOR (95% CI)	Model 3^c^ aOR (95% CI)	Model 4^d^ aOR (95% CI)
All women					
**hsCRP (mg/L), AHA recommended categories**					
Tertile 1 (0.1–1.0)	53/144	Ref.	Ref.	Ref.	Ref.
Tertile 2 (1.0–3.0)	75/163	1.46 (0.93–2.31)	1.45 (0.92–2.30)	1.50 (0.89–2.52)	1.61 (0.91–2.84)
Tertile 3 (> 3.0)	168/248	**3.61 (2.34–5.55)**	**3.60 (2.34–5.54)**	**4.46 (2.70–7.38)**	**4.43 (2.56–7.66)**
^*^Continuous hsCRP (**mg/L**)	296/555	**1.30 (1.20–1.40)**	**1.30 (1.20–1.40)**	**1.31 (1.20–1.43)**	**1.31 (1.19–1.45)**
Pre-/Peri-Menopause					
**hsCRP (mg/L), AHA recommended categories**					
Tertile 1 (0.1–1.0)	30/70	Ref.	Ref.	Ref.	Ref.
Tertile 2 (1.0–3.0)	32/67	1.22 (0.62–2.39)	1.27 (0.65–2.51)	1.13 (0.52–2.47)	1.19 (0.50–2.66)
Tertile 3 (> 3.0)	81/115	**3.18 (1.71–5.91)**	**3.26 (1.74–6.10)**	**4.64 (2.22–9.72)**	**4.69 (2.10–10.51)**
^*^Continuous hsCRP (**mg/L**)	143/252	**1.26 (1.13–1.40)**	**1.26 (1.13–1.40)**	**1.32 (1.16–1.49)**	**1.34 (1.16–1.56)**
Post-Menopause					
**hsCRP (mg/L), AHA recommended categories**					
Tertile 1 (0.1–1.0)	23/69	Ref.	Ref.	Ref.	Ref.
Tertile 2 (1.0–3.0)	43/88	1.91 (1.00–3.67)	1.88 (0.98–3.61)	1.95 (0.94–4.05)	2.07 (0.95–4.53)
Tertile 3 (> 3.0)	87/127	**4.35 (2.33–8.13)**	**4.35 (2.33–8.14)**	**4.48 (2.24–8.98)**	**4.34 (2.05–9.22)**
^*^Continuous hsCRP (**mg/L**)	153/284	**1.33 (1.18–1.50)**	**1.33 (1.18–1.50)**	**1.31 (1.15–1.49)**	**1.29 (1.12–1.48)**

^*^Continuous values of hsCRP are log_2_-transformed. ^a^Model 1, unadjusted; ^b^Model 2, adjusted for age; ^c^Model 3, additionally adjusted for reproductive characteristics: age at menarche, number of pregnancies, number of births, and menopausal status (not included in the stratified models); ^d^Model 4, additionally adjusted for BMI, diabetes and hypertension status; Bolded values indicate significance at *p* < 0.05. Abbreviations: OR, odds ratio; CI, confidence interval; aOR, adjusted odds ratio. *n* = Number of breast cancer cases within each category. *N* = Number of women in each category.

**Table 3:** Multivariable associations of hsCRP and breast cancer by BMI category

	n/N	Model 1^a^ OR (95% CI)	Model 2^b^ aOR (95% CI)	Model 3^c^ aOR (95% CI)	Model 4^d^ aOR (95% CI)
Normal weight					
**hsCRP (mg/L), AHA recommended categories**					
Tertile 1 (0.1–1.0)	26/67	Ref.	Ref.	Ref.	Ref.
Tertile 2 (1.0–3.0)	27/55	1.52 (0.74–3.13)	1.44 (0.69–3.00)	1.44 (0.59–3.50)	1.51 (0.59–3.88)
Tertile 3 (> 3.0)	67/91	**4.40 (2.24–8.67)**	**4.65 (2.33–9.27)**	**5.02 (2.12–11.90)**	**5.72 (2.22–14.74)**
^*^Continuous hsCRP (**mg/L**)	120/213	**1.31 (1.17–1.47)**	**1.32 (1.00–1.05)**	**1.30 (1.12–1.50)**	**1.31 (1.12–1.54)**
Overweight					
**hsCRP (mg/L), AHA recommended categories**					
Tertile 1 (0.1–1.0)	15/42	Ref.	Ref.	Ref.	Ref.
Tertile 2 (1.0–3.0)	26/52	1.80 (0.78–4.14)	1.79 (0.78–4.12)	2.05 (0.77–5.44)	2.21 (0.77–6.31)
Tertile 3 (> 3.0)	47/73	**3.25 (1.47–7.19)**	**3.34 (1.50–7.41)**	**4.96 (1.91–12.89)**	**4.11 (1.47–11.51)**
^*^Continuous hsCRP (**mg/L**)	88/167	**1.29 (1.11–1.49)**	**1.29 (1.11–1.50)**	**1.32 (1.11–1.56)**	**1.29 (1.06–1.55)**
Obese					
**hsCRP (mg/L), AHA recommended categories**					
Tertile 1 (0.1–1.0)	7/29	Ref.	Ref.	Ref.	Ref.
Tertile 2 (1.0–3.0)	16/48	1.57 (0.55–4.45)	1.49 (0.52–4.25)	1.63 (0.51–5.25)	1.39 (0.41–4.69)
Tertile 3 (> 3.0)	42/69	**4.89 (1.84–13.00)**	**4.89 (1.83–13.03)**	**6.32 (2.09–19.08)**	**5.35 (1.72–16.62)**
^*^Continuous hsCRP (**mg/L**)	65/146	**1.56 (1.26–1.93)**	**1.58 (1.27–1.96)**	**1.65 (1.29–2.12)**	**1.65 (1.28–2.13)**

^*^Continuous values of hsCRP are log_2_-transformed. ^a^Model 1, unadjusted; ^b^Model 2, adjusted for age; ^c^Model 3, additionally adjusted for reproductive characteristics: age at menarche, number of pregnancies, number of births, and menopausal status (not included in the stratified models); ^d^Model 4, additionally adjusted for BMI, diabetes and hypertension status; Bolded values indicate significance at *p* < 0.05. Abbreviations: OR, odds ratio; CI, confidence interval; aOR, adjusted odds ratio. *n* = Number of breast cancer cases within each category. *N* = Number of women in each category.

In multinomial logistic regression models evaluating the odds of BC molecular subtypes compared with controls (Table 4), high hsCRP (> 3.0 mg/L) was statistically significantly associated with increased odds of luminal A (aOR: 4.03; 95% CI: 1.29, 12.64), TN (aOR: 3.32; 95% CI: 1.07, 10.35), and HER2-enriched (aOR: 6.27; 95% CI: 1.69, 23.25) BC subtypes. Doubling of hsCRP concentration was associated with 23%, 19%, 30% and 39% increased odds of luminal A, luminal B, TN, and HER2-enriched BC subtypes, respectively, although the association was not statistically significant for the luminal B subtype. In sensitivity analyses excluding women with hsCRP values > 10 mg/L, patterns of association were largely consistent with overall analyses (data not shown).

**Table 4:** Associations of hsCRP and breast cancer subtype

	Luminal A aOR (95% CI)	Luminal B aOR (95% CI)	Triple Negative aOR (95% CI)	HER2 aOR (95% CI)
**hsCRP (mg/L)**				
Tertile 1 (0.1–1.0)	Ref.	Ref.	Ref.	Ref.
Tertile 2 (1.0–3.0)	1.14 (0.30–4.29)	0.99 (0.32–3.07)	2.52 (0.80–7.97)	1.67 (0.37–7.21)
Tertile 3 (> 3.0)	**4.03 (1.02–12.64)**	1.80 (0.61–5.29)	**3.32 (1.07–10.35)**	**6.27 (1.69–23.25)**
^*^Continuous hsCRP (**mg/L**)	**1.23 (1.02–1.49)**	1.19 (0.99–1.43)	**1.30 (1.09–1.55)**	**1.39 (1.15–1.69)**

^*^Continuous values of hsCRP are log_2_-transformed. Multinomial logistic regression models predicted odds of specific breast cancer subtype versus controls. Adjusted for reproductive characteristics: age at menarche, number of pregnancies, number of births, and menopausal status, BMI, hypertension and diabetes status. Bolded values indicate significance at *p* < 0.05. Abbreviations: aOR, adjusted odds ratio; CI, confidence interval.

## DISCUSSION

In the first analysis of hsCRP among newly diagnosed BC cases and healthy controls in Nigeria, we observed that cases were significantly more likely to have high hsCRP compared with controls. After adjusting for socio-demographic, clinical, and reproductive variables, high hsCRP was associated with a statistically significant 4-fold increased odds of BC, an association that remained significant regardless of menopausal status and BMI category. We also provide novel evidence of associations between hsCRP and BC molecular subtypes, with significant associations observed for luminal A, TN, and HER-enriched subtypes.

These findings are consistent with several studies utilizing case-control and prospective cohort study designs in the United States, Europe, and Asia, as well as systematic reviews that have reported an increased risk of BC with elevated levels of CRP [14, 15, 22–25]. However, other studies have reported conflicting results and have failed to find a significant positive association [12, 26, 27]. One of these reviews noted that although most prevalent case-control or cross-sectional studies found higher CRP concentrations in cancer patients compared to healthy controls, prospective cohort studies provided no strong evidence for an association [12]. In addition to study design differences, the conflicting results may also be due to characteristics of study populations. For instance, a prospective cohort study in Europe noted no association between CRP levels and BC risk among postmenopausal women overall but observed a statistically significant increase in BC risk among overweight and obese postmenopausal women [19]. This finding is inconsistent with our results of a positive association regardless of menopausal status or BMI category. Another prospective cohort study in China found that elevated levels of CRP at baseline were associated with an increased risk of BC overall and among younger women [28]. It is worth noting that the majority of past studies have been conducted in mostly White populations from the United States and Europe, and a few studies in Asia. Of those studies in the United States, no study to our knowledge has been specifically conducted among African American women despite notably higher CRP levels in this population [29]; ours is the first to characterize this association on the African continent.

We observed significant positive associations between hsCRP levels and odds of luminal A, TN, and HER2-enriched BC. Past research by molecular subtype is extremely limited. One study in Italy reported a positive association between high CRP and TN and luminal B premenopausal BC [20], while another study in China found that only hormone receptor positive and HER2 negative BC had elevated serum CRP [21]. We urge further epidemiological studies on this topic among diverse populations to better characterize the association of CRP with BC risk by molecular subtype.

Several reasons may explain our observed positive association between hsCRP levels and odds of BC. It has been well documented that systemic and chronic inflammation is induced by obesity, alcohol consumption, and other poor lifestyle factors, leading to the development of cancer via the secretion of pro-inflammatory molecules and the dysregulation of physiological processes such as oxidative stress mechanisms and autophagy [30–32]. The inflammatory response can also induce genetic changes, such as mutations in tumor suppressor genes, leading to the development and progression of cancer [32]. For example, in the context of obesity, inflammatory mediators are released in response to the excessive accumulation of macronutrients in adipose tissue in order to maintain homeostasis [31], and regular use of nonsteroidal anti-inflammatory drugs is associated with reduced BC risk [33]. However, because we did not record the use of these drugs in our study, we were unable to examine this association in the present analysis. Underlying infections, highly prevalent in the Nigerian context, may also induce systemic inflammation, although there is no data suggesting an association between infections and BC among Nigerian women. However, chronic stress due to a recent diagnosis of BC may increase systemic inflammation, potentially explaining our findings. Future studies in this population may help to clarify the biological mechanisms linking CRP with BC by molecular subtype.

Stromal cells which pertain to the tumor microenvironment (TME) have been shown to influence breast tumor progression [34–37]. These stromal cells are known as cancer-associated fibroblasts (CAF) and are thought to originate from tissue fibroblasts and mesenchymal stem cells (MSC) [38]. Further, adipose- and bone-marrow derived MSCs have been characterized as pro-cancerous [39–43]. Thus, the cellular makeup of the TME and its composition of inflammatory molecules and mediators collectively create an ecosystem that favors tumor growth [38, 44–48]. Indeed, it appears that tumor-stroma-inflammatory pathways may be responsible for the aggressive nature of TNBC [49], and thus may explain why we observed an association between hsCRP and odds of TNBC. Although we are unable to confirm the role of the TME in our data, future research regarding the TME in TNBC, especially among women of African descent, for whom TNBC is most prevalent [50], may help to unlock new targets for treatment and prevention of this most aggressive and recalcitrant molecular BC subtype.

There are several strengths and limitations of our study that are worth noting in the interpretation of results. First, several of the covariates included in this study were self-reported by participants, introducing potential recall bias in our analysis. However, our main exposure, hsCRP, was measured from blood samples simultaneously for cases and controls following the same protocol, minimizing misclassification. Second, it is unclear whether CRP levels cause BC, or are influenced by the presence of BC. Our study design limits our ability to rule out potential reverse causality as blood hsCRP levels were measured from blood samples obtained at the time of BC diagnosis. Third, we did not examine CRP levels directly in the breast tissue. This was beyond the scope of the current analysis but is worth exploring in future studies. Fourth, we acknowledge our sample size limitation for the molecular subtype analysis but believe that our study lays an important foundation for future large prospective studies among women of African descent. Despite these limitations, important strengths of our study include the use of histologically confirmed BC cases with data on BC molecular subtype and adjustment for BMI and menopausal status among other key covariates. Additionally, ours is the first study to characterize the association between CRP levels and BC risk among Nigerian women, and further to do so by cancer molecular subtype.

In conclusion, our analysis revealed a positive association between hsCRP and odds of BC, overall and for all molecular subtypes. Because CRP is an easily measured biomarker in the blood, it may represent a useful predictor of BC in the Nigerian context. We urge larger studies, preferably prospective cohort studies, among women of African descent to further characterize this association.

## MATERIALS AND METHODS

### Study design

We have previously described The Mechanisms for Established and Novel Risk Factors for Breast Cancer in Women of African Descent (MEND) study in detail [51]. Briefly, newly diagnosed BC patients from four hospitals in southwestern Nigeria were enrolled in the MEND study between 2015 and 2019. A trained nurse explained the requirements of the study to BC patients during their clinical visits, and participants who expressed interest in participating were evaluated for eligibility. Patients were excluded if they were unable to communicate in English to complete the baseline survey or if they had other medical conditions that could affect participation. Upon providing verbal and written informed consent, study participants completed a questionnaire related to their sociodemographic characteristics, reproductive history, and past personal and family history of cancer. Next, anthropometric measurements were taken, and a blood sample and tumor biopsy sample were collected. All blood samples were obtained prior to surgery or chemotherapy treatment. Tissue and blood samples were processed and stored in -80°C freezers until shipment to the United States for additional analysis. Participants received a N500 telephone recharge card (valued at US $1.50) in addition to the supplies necessary for their biopsies for their participation in this study. Healthy controls with BC were selected from a cohort of 4,000 women recruited from Nigeria and Ghana as part of the Human Heredity and Health (H3) Africa Chronic Kidney Disease (CKD) Study [52]. H3Africa recruitment overlapped with case recruitment and occurred between 2015 and 2017. It involved the recruitment of healthy, community-based adult women, and the collection of extension socio-demographic, clinical, family history and behavioral risk factor data. Controls also provided blood samples. Sample and data collection protocols were similar between the CKD and MEND studies. Biospecimen for cases and controls were assayed at the same time in the same laboratory with the laboratory technician blinded to case status. These procedures were approved by the Institutional Review Boards at Duke University and the participating hospitals.

### Breast cancer cases and subtyping

BC cases were determined in one of two ways, either from pathology reports of clinical biopsy samples evaluated by a trained pathologist from the diagnosing hospital in Nigeria, or from samples that were shipped to the United States and evaluated by a pathologist. The sample was considered a confirmed case if either report indicated a cancer diagnosis. These samples underwent immunohistochemistry as part of regular standard of care procedures in Nigeria or at the Duke University BioRepository and Precision Pathology Center. United States typing was used if results from both sources were available, as it constituted most of the available immunohistochemistry information. The Allred method was used to score estrogen receptor (ER) and progesterone receptor (PR) status [53, 54]. Stain intensity was categorized as 0 (none), 1 (mild), 2 (moderate), or 3 (strong). The proportion of nuclear positivity was defined as 0 (0%), 1 (< 1%), 2 (1–10%), 3 (11–33%), 4 (33–66%) or 5 (67–100%). ER/PR status was categorized as positive (3–8) or negative (0–2). HER2 status was defined as negative (scores = 0–1) or positive (score = 3) [55]. There were no equivocal (score = 2) results in our sample. From these categorizations of hormone receptor status, BC molecular subtype was determined: luminal A (ER+ and/or PR+ / HER2-), luminal B (ER+ and/or PR+ / HER2+), TN (ER- / PR- / HER2-), or HER2 (ER- / PR- / HER2+). In total, 124 cases had data available on ER/PR/HER2 status and were classified into a molecular subtype.

### CRP and study covariates

Biospecimens for confirmed BC cases with completed surveys were submitted to the Duke Molecular Physiology Institute Immunoassay laboratory for analysis and tested for hsCRP. These measurements were performed using a latex-enhanced immunoturbidimetric assay from Beckman (Brea, CA, USA) on a Beckman DxC 600 clinical analyzer. BMI (kg/m^2^) was calculated from height and weight which were collected by trained research staff at enrollment. Reproductive and clinical characteristics, including age at menarche, number of pregnancies and births, menopausal status, prior diabetes, and hypertension diagnosis were self-reported by participants. Participants who self-reported a history of cancer or missing personal cancer history were excluded from analysis. Missing data for all variables were not used for statistical analyses.

### Statistical analysis

hsCRP was categorized into < 1.0 mg/L, 1.0–3.0 mg/L and > 3.0 mg/L according to the American Heart Association and U.S Centers for Disease Control and Prevention guidelines [56]. Differences in demographic, clinical and reproductive characteristics were compared between cases and controls as well as by hsCRP categories using Wilcoxon rank sum/Kruskal-Wallis tests for continuous variables and χ^2^ (chi-squared) tests for categorical variables. Univariable and multivariable logistic regression models were used to test the association between hsCRP (separately assessed as categorical and continuous (log_2_-transformed)) and BC diagnosis. Estimates for the log_2_-transformed hsCRP are interpreted as the relative risk associated with a doubling in hsCRP concentration [57]. Multivariable models were adjusted for (a) age at enrollment only, (b) age at enrollment, age at menarche, number of pregnancies (categorized as < 4 vs. ≥ 4), number of births (categorized as < 4 vs. ≥ 4) and menopausal status and (c) in addition to variables in (b), BMI, hypertension, and diabetes. Hypertension status was self-reported among cases and controls. Among cases, women with fasting-glucose levels of > 100 mg/dL were classified as diabetic while among controls, diabetes status was self-reported. These models were repeated with stratification for menopausal status and BMI categories (Normal weight, Overweight and Obese). Furthermore, among BC cases with cancer subtyping data available, odds of luminal A, luminal B, TN, or HER2 cancer subtypes were compared to controls using multinomial logistic regression models. In sensitivity analyses, we repeated all analyses after excluding women with hsCRP values > 10 mg/L. There was no statistically significant difference in hsCRP values between controls recruited in Nigeria and Ghana (*p* = 0.3363), therefore we present overall results. All statistical significance tests were two-sided with *p* < 0.05 defined as significant. Statistical analyses were conducted using SAS Version 9.4 (SAS institute, Cary, NC, USA).

### Ethics statement

The study was conducted according to the guidelines of the Declaration of Helsinki and approved by the Institutional Review Board of Duke University (protocol code Pro00102004, approved 5/17/2019). Informed consent was obtained from all subjects involved in the study.

## SUPPLEMENTARY MATERIALS





## Abbreviations

95% CI: 95% confidence interval
aOR: adjusted odds ratio
BMI: body mass index
BC: breast cancer
CAF: cancer-associated fibroblast
CKD: chronic kidney disease
CRP: C-reactive protein
ER: estrogen receptor
HER2: human epidermal growth factor-2
H3A: Human Heredity and Health Africa
hsCRP: high-sensitivity C-reactive protein
MEND: Mechanisms for Established and Novel Risk Factors for Breast Cancer in Women of African Descent
MSC: mesenchymal stem cell
OR: odds ratio
PR: progesterone receptor
TME: tumor microenvironment
TN: triple-negative

## Notes

**Editorial note**

This paper has been accepted based in part on peer-review conducted by another journal and the authors' response and revisions as well as expedited peer-review in Oncotarget.
