# Supplementary material for: Association of high-sensitivity C-reactive protein and odds of breast cancer by molecular subtype: analysis of the MEND study
Source: Oncotarget. 2021 Jun 22;12(13):1230–42. doi: 10.18632/oncotarget.27991 (PMC8238238; doi:10.18632/oncotarget.27991)
Supplement: Supplementary file 1 [file oncotarget-12-1230-s001.pdf]

Association of high-sensitivity C-reactive protein and odds of breast cancer by molecular subtype: analysis of the MEND study

SUPPLEMENTARY MATERIALS

Supplementary Table 1: Clinical and reproductive characteristics by case/control status stratified by AHA-defined hsCRP categories. See Supplementary Table 1
