# Supplementary material for: Association of high-sensitivity C-reactive protein and odds of breast cancer by molecular subtype: analysis of the MEND study
Source: Oncotarget. 2021 Jun 22;12(13):1230–42. doi: 10.18632/oncotarget.27991 (PMC8238238; doi:10.18632/oncotarget.27991)
Supplement: Supplementary file 2 [file oncotarget-12-1230-s002.docx]

| **Supplementary Table 1: Clinical and reproductive characteristics by case/control status stratified by AHA-defined hsCRP categories** | | | | | | | | | |
| --- | --- | --- | --- | --- | --- | --- | --- | --- | --- |
|  | **hsCRP (mg/L)** | | | | | | | | |
|  | **(0.1-1.0)**  **(*N* = 144)** | | | **(1.0-3.0)**  **(*N* = 163)** | | | **> 3**  **(*N* = 248)** | | |
|  | **Case (*N* = 53)** | **Control (*N* = 91)** | ***p* value** | **Case (*N* = 75)** | **Control (*N* = 88)** | ***p* value** | **Case (*N* = 168)** | **Control (*N* = 80)** | ***p* value** |
| Demographics |  |  |  |  |  |  |  |  |  |
| Age (years)^a^ | 47 (32-77) | 46 (27-74) | 0.982^1^ | 51 (30-82) | 49 (26-72) | 0.907^1^ | 49 (23-85) | 48 (18-70) | 0.481^1^ |
| Clinical characteristics |  |  |  |  |  |  |  |  |  |
| BMI |  |  | 0.104^2^ |  |  | 0.198^2^ |  |  | 0.337^2^ |
| Underweight | 4 (7.5%) | 1 (1.1%) |  | 4 (5.3%) | 2 (2.3%) |  | 8 (4.8%) | 3 (3.8%) |  |
| Normal weight | 26 (49.1%) | 41 (45.1%) |  | 27 (36.0%) | 28 (31.8%) |  | 67 (39.9%) | 24 (30.0%) |  |
| Overweight | 15 (28.3%) | 27 (29.7%) |  | 26 (34.7%) | 26 (29.5%) |  | 47 (28.0%) | 26 (32.5%) |  |
| Obese | 7 (13.2%) | 22 (24.2%) |  | 16 (21.3%) | 32 (36.4%) |  | 42 (25.0%) | 27 (33.8%) |  |
| Height, cm^a^ | 62.8 (59.1-70.1) | 63.0 (56.7-69.3) | 0.670^1^ | 63.7 (57.5-69.3) | 63.0 (51.8-69.5) | 0.435^1^ | 63.2 (56.1-69.5) | 62.5 (58.7-68.2) | 0.188^1^ |
| Weight, kg^a^ | 138.0 (93.5-206.0) | 143.3 (78.9-226.5) | 0.016^1^ | 148.8 (82.0-255.2) | 153.8 (91.5-259.3) | 0.318^1^ | 143.0 (81.6-228.4) | 150.1 (85.8-289.7) | 0.139^1^ |
| Systolic BP^a^ | 121.7 (94.7-167.3) | 124.7 (91.0-192.7) | 0.266^1^ | 130.0 (84.7-196.0) | 127.7 (77.7-188.0) | 0.449^1^ | 125.0 (84.0-236.0) | 129.8 (95.3-231.3) | 0.122^1^ |
| Diastolic BP^a^ | 74.0 (53.3-104.0) | 76.7 (52.0-116.0) | 0.463^1^ | 83.3 (41.0-113.7) | 76.0 (52.0-128.7) | 0.007^1^ | 78.8 (47.3-136.0) | 77.8 (35.3-126.3) | 0.521^1^ |
| Prior diabetes diagnosis | 1 (1.9%) | 15 (16.5%) | 0.003^2^ | 1 (1.3%) | 11 (12.5%) | 0.002^2^ | 1 (0.6%) | 13 (16.3%) | < 0.001^2^ |
| Prior hypertension diagnosis | 8 (15.1%) | 40 (44.0%) | < 0.001^2^ | 13 (17.3%) | 46 (52.3%) | < 0.001^2^ | 35 (20.8%) | 39 (48.8%) | < 0.001^2^ |
| Reproductive history |  |  |  |  |  |  |  |  |  |
| Age at menarche^a^ | 15 (11-22) | 15 (11-21) | 0.042^1^ | 15 (10-21) | 15 (10-28) | 0.221^1^ | 15 (9-21) | 15 (11-21) | 0.150^1^ |
| Ever pregnant | 47 (88.7%) | 87 (95.6%) | 0.023^2^ | 70 (93.3%) | 83 (94.3%) | 0.355^2^ | 165 (98.2%) | 73 (91.3%) | 0.137^2^ |
| Number of pregnancies^a,b^ | 4 (1-9) | 4 (1-12) | 0.877^1^ | 4 (1-11) | 5 (1-14) | 0.836^1^ | 5 (1-11) | 5 (1-12) | 0.752^1^ |
| Number of births^a,b^ | 4 (0-9) | 3 (0-16) | 0.676^1^ | 4 (0-9) | 4 (0-10) | 0.882^1^ | 4 (0-10) | 4 (0-10) | 0.818^1^ |
| Menopausal status |  |  | 0.248^2^ |  |  | 0.892^2^ |  |  | 0.745^2^ |
| Pre- or peri-menopause | 30 (56.6%) | 40 (44.0%) |  | 32 (42.7%) | 35 (39.8%) |  | 81 (48.2%) | 34 (42.5%) |  |
| Post-menopause | 23 (43.4%) | 46 (50.5%) |  | 43 (57.3%) | 45 (51.1%) |  | 87 (51.8%) | 40 (50.0%) |  |
| Ever used HRT | 0 (0.0%) | 13 (14.3%) | 0.001^2^ | 0 (0.0%) | 9 (10.2%) | 0.001^2^ | 2 (1.2%) | 17 (21.3%) | < 0.001^2^ |
| ^1^Wilcoxon rank sum test, ^2^Chi-Square test.  ^a^Median (range); ^b^Among those who were ever pregnant.  Where applicable, missing values were not used in generating *p*-value. | | | | | | | | | |
